# Transient line starting analysis of the ultra-high speed PMSM

**DOI:** 10.1063/1.4974057

**Published:** 2017-01-11

**Authors:** Wenjie Cheng, Wei Li, ling Xiao, Ming Li, Yongsheng Tian, Yanhua Sun, Lie Yu

**Affiliations:** 1 Department of Basic Courses, Mechanics Research Center, Xi’an University of Science and Technology, Xi’an, Shaanxi 710054, China; 2 Institute of Engineering Thermophysics, Chinese Academy of Science Corporation or Laboratory, No. 11, Beisihuanxi Road, 100190, Beijing, China; 3 Institute of Mechatronics and Information Systems, State Key Laboratory for Strength and Vibration of Mechanical Structures, Xi’an Jiaotong University, Xi’an, 710049, China

## Abstract

Aiming at the ultra high speed permanent magnet synchronous motor (PMSM) supported by gas foil bearings (GFBs), this paper calculates the transient line starting of the motor. Firstly, the start effect of the rotor composed of cylindrical PM and stainless steel sleeve is studied. Then, in order to enhance the start torque, copper ring, nickel ring and copper squirrel-cage are introduced in the rotor and their start effect are analysed, respectively. It can be found that the rotor including nickel ring can be accelerated to set speed, but all the other rotors are failed due to the higher PM and braking torques. It can be concluded that some material owning slight large relative permeability can be applied in the rotor to reduce the PM field and contribute to start by using the line-start method.

## I. INTRODUCTION

In spite of the superior performance of the PMSM supported by GFBs, the start is difficult due to two factors, one is the dry friction torque from the preload GFBs, and the other is the lack of the starting winding in the rotor. As the starting winding cannot be imbedded in the rotor, the motor is not suited to asynchronous starting. Generally, the frequency converter combined with the rotor position closed loop control system can realize synchronous starting, however, the system is of high cost and limited to ultra high speed.

Actually, the ultra-high speed PM rotors adopt a little permeability, high conductivity metallic sleeve, which equals to a set of damping windings (infinite conducting bar in circumferential direction). This structure indicates the PMSMs can start by using the eddy currents in the metallic sleeve. The literature^1^ carries out an earlier study on the starting of PMSM using eddy currents, and applies an copper ring in the canned rotor to increase the starting torque. However, their PMSM rated speed is low (3000r/min), and stator slot effects as well as winding distribution are not considered. When the PM is not magnetized, the PMSM will become the solid rotor induction motor. This kind of motor is studied in literatures^2–3^ where stator winding current is simplified as a sinusoidal fundamental current sheet, but stator slot effects and high order harmonic current are ignored. Literature^4^ calculates rotor eddy losses analytically by using double exponential series, but neglects stator slot effects and PM field. Indeed, a magnetized PM rotates in the stator bore can also generate eddy losses and braking torque.^5^ Now the complex relative air-gap permeance^6–7^ and subdomain methods^8–10^ can be used to describe the stator slot effects analytically, but they are not very suitable for transient calculation. In addition, the eddy currents reaction field has a great influence on the air gap field and can further affect the electromagnetic torque. Gas foil bearings need preload in order to enhance the stability of the bearing-rotor systems,^11^ so dry friction torque will be very dramatic. Therefore, it is significant to check the starting performance of the PMSMs.

In order to study the starting performance of the ultra-high speed PMSMs by using the line starting method, the paper carries out a transient starting simulation considering the stator slot effects and eddy currents reaction field by using field-circuit coupling finite-element method (FEM), aiming at a 13kW, 120,000rpm PMSM, and makes experiment as well. The starting performance of the PMSM with different rotors is studied and compared, and some conclusions are summarized.

## II. MODEL OF THE PMSM

**FIG. 1.**
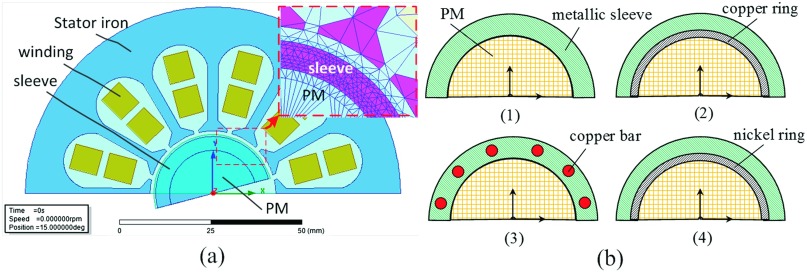
The PMSM FEM model and four type of ultra-high speed PM rotors.

The field-circuit coupling FEM model of the ultra high speed PMSM feeding by three phase high frequency AC voltage is shown in Figure 1(a).

Figure 1(b1) shows the original rotor in which a metallic sleeve (e.g. inconel718, Ti-6Al-4V) is interference fitted onto a cylindrical PM. In order to enhance the pull-in torque, copper ring and copper bar are implied as shown in Figure 1(b2) and Figure 1(b3) respectively. In addition, materials with low resistivity and slight high relative permeability such as nickel ring are considered as shown in Figure 1(b4). Rotor temperature and strength are not considered in the paper. Parameters of the rotors and the motor are listed in Table I where resistivity and relative permeability are denoted by *ρ* and $\mu_{r}$, respectively.

**TABLE I.** Parameters of various rotors and PMSM.

Rated voltage	Rated current	Rated torque	Supply voltage	Supply frequency	Air gap flux density
300V_rms_/line	24A_rms_	1.0Nm	135V/phase	1000Hz	0.33T_ave_
Copper ring *ρ*	Copper ring $\mu_{r}$	Nickel ring *ρ*	Nickel ring $\mu_{r}$	Stainless steel *ρ*	Stainless steel $\mu_{r}$
$17\times 10^{-9}\Omega \text{m}$	1	$69\times 10^{-9}\Omega \text{m}$	600	$910\times 10^{-9}\Omega \text{m}$	1

## III. RESULTS AND ANALYSES

### A. Simulation results

**FIG. 2.**
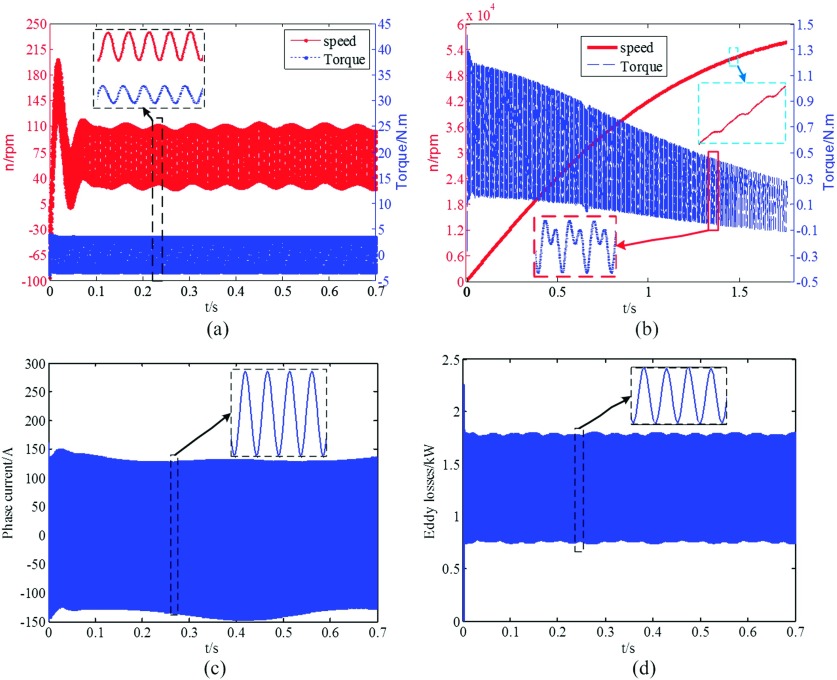
Transient performance of the PMSM by using the line starting method.

When the motor adopts the copper rotor and is powered by 135V, 1000Hz AC voltage, the results are shown in Figure 2(a) in which the speed cannot go up but fluctuates as a center 70r/min with an amplitude 35r/min and the transient starting torque waves in ±4Nm. The corresponding transient phase current (92A_rms_) and rotor eddy losses (average value 1.25kW) are also fluctuating as shown in Figure 2(c) and Figure 2(d) respectively. The starting performances of the motor with the original and copper bar rotors are similar to that with the copper ring rotor (their speeds are still low and fluctuated). The cause of the above phenomenon is the PM torque (generated by the interaction between the armature field and the PM field) and the generating braking torque (generated by windings under the magnetic field) are much larger than the induction torque due to rotor eddy currents. In the starting period, the armature field does the circular motion relative to the PM, in a half cycle, the PM torque works in drive mode to accelerate the rotor, but in the other half, it works in braking mode to decelerate the rotor. Therefore, the fluctuation will appear in the speed curve as well as in the loss curve.

According to the above discussion, nickel ring rotor is applied to reducing the PM field while can still offer high eddy currents simultaneously, and results are shown in Figure 2(b). It can be found the rotor can achieve 56,000r/min at 1.7s with a slight ripple, the average value and the vibration amplitude of the electromagnetic torque decrease with the increase of speed and will eventually tend to a constant value. As to the electromagnetic torque of the copper ring rotor, the leading component is the PM torque as shown in Figure 2(a), and its amplitude is about 4Nm which is almost 5 times larger than the rated torque. While as to the electromagnetic torque of the nickel ring rotor, its average (the combination of the induction torque and the generating braking torque) falls from 0.7Nm to 0.1Nm over time, and its vibration amplitude (the PM torque) decreasing from 0.6Nm to 0.2Nm over time. Therefore, it can be concluded that the induction torque minus by the generating braking torque should be comparable to the PM torque to guarantee that the rotor can be successfully pulled into the synchronous speed in a short time such as 5s. In fact, the PM rotor will degrade as the solid rotor completely when the sleeve uses iron alloy $\left(\mu_{r}=4000\right)$ and the corresponding pull-in time is about half of the nickel ring’s.

**FIG. 3.**
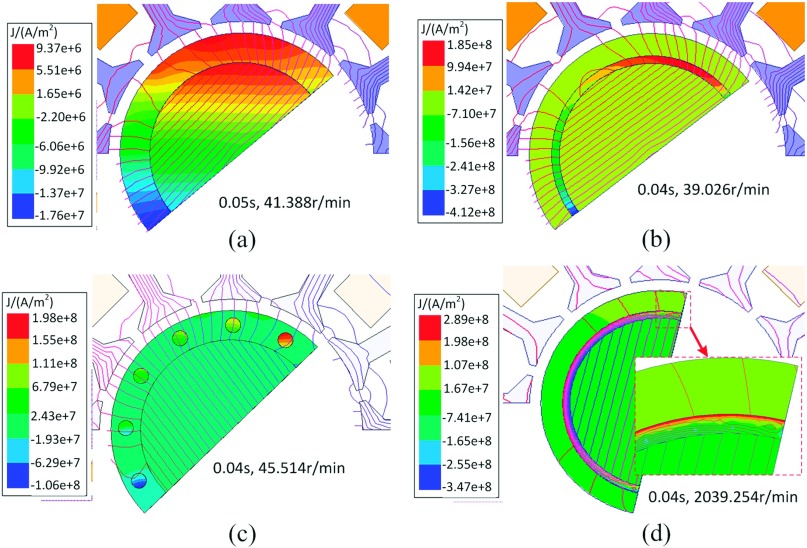
Eddy currents and magnetic fields of four types of rotor.

Figure 3(b) shows the case equipped with copper ring, in 0.04 seconds, the fundamental component amplitude of air gap flux density is about 0.575T, and the corresponding average value is 0.36T which is slight larger than the design value. However, for the case equipped with nickel ring, the average value is 0.19T which is 56% of the design value at the same time, that is because the nickel ring $\left(\mu_{r}=600\right)$ absorbs a large proportion of PM field. In addition, Figure 3(a) shows that the original rotor has a wide eddy currents region because the stainless steel has a large resistivity and its skin depth is 7 times of the copper’s (2.1mm). Figure 3(b) shows eddy currents are mainly concentrated in the copper ring, and the eddy currents amplitude is about 19 times larger than that of original rotor, but the induction torque generated by the copper ring is still insufficient because of its small cross sectional area. The results of copper bar rotor are shown in Figure 3(c), in 0.04 seconds, the eddy currents amplitude is almost equal to that of copper ring rotor, and the speed (45.514r/min) is a little bigger than the latter (39.026r/min), unfortunately, the copper bar rotor also cannot be accelerated continuously. In Figure 3(d), the motor applies the nickel ring rotor, the speed has arrived 2039.254r/min at 0.04 seconds, eddy currents mainly concentrate on the rotor outer surface and its amplitude is 1.56 times larger than that of the copper ring. Notably, about half of the PM field is shielded by the nickel ring.

### B. Experiment

**FIG. 4.**
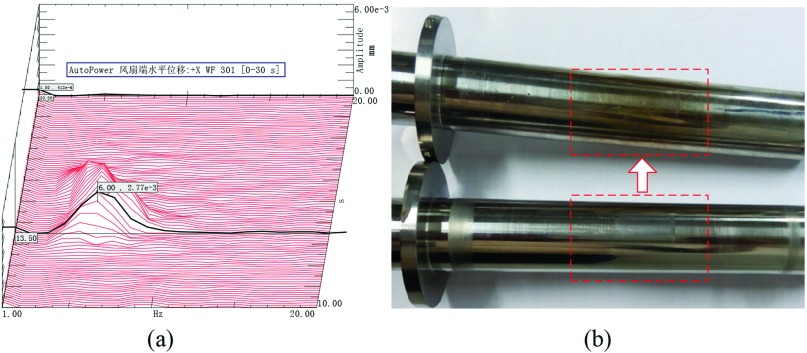
Waterfall plot of the experiment and original rotors.

Start-up experiment with the original rotor has been carried out where a 1000Hz, 300Vrms AC line voltage is applied to overcome the dry friction torque, and the results are shown in Figure 4. It shows that the maximum speed is about 360r/min with the only 4s run time. After the experiment, the rotor has been completely demagnetizated, and yellow spot appears as shown in Figure 4(b). In the open loop V/F start-up stage, the power supply voltage and the winding current are linearly rising. The increasing winding current will further enhance the rotor eddy currents, and the rotor temperature will rise quickly without insufficient cooling. In fact, the resistivity of the sleeve will become larger with the increase of temperature, for example, the resistivity of Inconel718 is $1.25\times 10^{-6}\Omega .\text{m}$ at 20°C but $7.53\times 10^{-6}\Omega .\text{m}$ at 700°C. Whereupon, rotor eddy currents and corresponding induction torque at 350°C (demagnetization temperature of NbFeB) are only 28.5% of that at 20°C, respectively. If the rotor temperature continue to rise, the induction torque will further decline, then the rotor speed will drop to zero under the action of dry friction and braking torques.

## IV. DISCUSSION AND CONCLUSION

The paper studies the transient line starting of the ultra-high speed PMSM, research shows that the original, copper ring and copper bar rotors all cannot be pulled into a high synchronous speed but fluctuate at a low speed, and the rotor eddy currents are extremely vigorous. It is because that the PM field of the above rotors is so intensive that the PM torque and the generating braking torque are much larger than the induction torque. Therefore, nickel ring rotor is applied, and the rotor can achieve 56,000r/min at 1.7s with a slight ripple. It can be concluded that the induction torque minus by the generating braking torque should be comparable to the PM torque to guarantee that the rotor can be successfully pulled into the synchronous speed over a short time.

Although the line starting method is feasible by introducing the nickel ring, the air gap flux density is weakened, which causes the motor power decreasing. In addition, in the speed-up stage, the rotor eddy losses is highly serious, which brings a great challenge to rotor cooling. However, it can give us the following inspirations: (1) some types of special materials which have large permeability at low temperature but small permeability at high temperature can be used in the ultra-high speed PM motor, that is, when the motor starts at room temperature, the PM field will be absorbed by the materials contributing to a low air gap flux density, then the induction torque accelerates the rotor, and when the motor works steadily in a high temperature, the materials reduce their permeability, then the PM field is released and PM torque predominates. (2) hysteresis ring which can generate a driven hysteresis torque maybe a feasible choice to assist starting, and some effective measures should be taken to solve the rotor cooling as well.
